# Efficient Overproduction of Active Nitrile Hydratase by Coupling Expression Induction and Enzyme Maturation *via* Programming a Controllable Cobalt-Responsive Gene Circuit

**DOI:** 10.3389/fbioe.2020.00193

**Published:** 2020-03-24

**Authors:** Laichuang Han, Wenjing Cui, Qiao Lin, Qiaoqing Chen, Feiya Suo, Ke Ma, Yang Wang, Wenliang Hao, Zhongyi Cheng, Zhemin Zhou

**Affiliations:** Key Laboratory of Industrial Biotechnology, School of Biotechnology, Jiangnan University, Jiangsu, China

**Keywords:** expression system, nitrile hydratase, cobalt-induced, metal homeostasis, protein expression

## Abstract

A robust and portable expression system is of great importance in enzyme production, metabolic engineering, and synthetic biology, which maximizes the performance of the engineered system. In this study, a tailor-made cobalt-induced expression system (CIES) was developed for low-cost and eco-friendly nitrile hydratase (NHase) production. First, the strong promoter P_veg_ from *Bacillus subtilis*, the Ni(II)/Co(II) responsive repressor RcnR, and its operator were reorganized to construct a CIES. In this system, the expression of reporter green fluorescent protein (GFP) was specifically triggered by Co(II) over a broad range of concentration. The performance of the cobalt-induced system was evolved to version 2.0 (CIES 2.0) for adaptation to different concentrations of Co(II) through programming a homeostasis system that rebalances cobalt efflux and influx with RcnA and NiCoT, respectively. Harnessing these synthetic platforms, the induced expression of NHase was coupled with enzyme maturation by Co(II) in a synchronizable manner without requiring additional inducers, which is a unique feature relative to other induced systems for production of NHase. The yield of NHase was 111.2 ± 17.9 U/ml using CIES and 114.9 ± 1.4 U/ml using CIES 2.0, which has a producing capability equivalent to that of commonly used isopropyl thiogalactoside (IPTG)-induced systems. In a scale-up system using a 5-L fermenter, the yielded enzymatic activity reached 542.2 ± 42.8 U/ml, suggesting that the designer platform for NHase is readily applied to the industry. The design of CIES in this study not only provided a low-cost and eco-friendly platform to overproduce NHase but also proposed a promising pipeline for development of synthetic platforms for expression of metalloenzymes.

## Introduction

Nitrile hydratase (NHase), a kind of metalloprotein catalyzing the hydration of a broad scope of nitriles to corresponding amides, has been widely used in chemical engineering to produce bulk chemicals and highly valuable medical intermediates, such as acrylamide, nicotinamide, and (*S*)-mandelamide (Kobayashi and Shimizu, 1998; Gong et al., 2017; Cheng et al., 2018; Supreetha et al., 2019). NHase is composed of α- and β-subunits, and according to the type of metals in the active site, it is classified into Fe-NHase and Co-NHase. Different from other metal proteins, NHase takes up metal ions under the help of an activator, and two Cys residues coordinated to the iron are posttranslationally modified to Cys-sulfenic and Cys-sulfinic acids (Nagashima et al., 1998). Engineered NHases that possess higher thermostability, activity, and stereoselectivity were also developed in various ways such as subunit fusion and semi-rational design (Xia et al., 2016; Cheng et al., 2017, 2018). In the industry, *Rhodococcus rhodochrous* J1 harboring NHase is widely used to produce acrylamide. However, the long cultivation time and low quantity of protein expression limit its application (Nagasawa et al., 1991; Zhou et al., 2008). Compared with *R. rhodochrous*, *Escherichia coli* is a more ideal host to efficiently produce NHase, due to its clear genetic background, fast growth, simple genetic manipulation, and strong ability of protein expression (Baneyx, 1999). To date, NHases from diverse organisms, such as *R. rhodochrous* J1 and *Pseudomonas putida*, have been heterologously expressed in *E. coli* (Xia et al., 2016; Lan et al., 2017), by which the cell-based catalytic technology using NHase has been established (Wang et al., 2017). However, the production of NHase by commonly used pET expression systems requires the addition of isopropyl thiogalactoside (IPTG), a kind of chemical to trigger the expression, which is an uneconomical platform to overproduce desired proteins at a large scale. Moreover, chemical inducers in these induced expression systems are unable to be eliminated in the culture medium, becoming a potential detriment to the environment.

The highly efficient, robust, and tunable gene expression system is essential for heterologous protein production (Ahmad et al., 2014; Ito et al., 2015; Marschall et al., 2016). Various gene expression systems are induced by physical signals (e.g., light and temperature), and natural and synthetic molecules have been constructed in different organisms (Tabor and Richardson, 1985; Bhavsar et al., 2001; Phan and Schumann, 2007; Zhao et al., 2018; Castillo-Hair et al., 2019). There are many determinants, such as the toxicity of inducers to cells, the catabolism of inducers, and the cost of inducers for scale-up production, that should be considered for choosing the most suitable expression system to the production of specific proteins. In respect to the studies focusing on enzymology and synthetic biology, the universal expression system pET system induced by IPTG is still the most widely used platform to mediate heterologous expression (Wang et al., 2018), which limits the development of sophisticated gene circuits requiring regulation by tailor-made expression systems responding to distinct inducers. Therefore, the tunable expression system orthogonal to metabolites is ideal for that goal (Bervoets et al., 2018; Meyer et al., 2019).

In *E. coli*, the *rcn* operon is involved in the homeostasis of cobalt and nickel ions (Iwig et al., 2006; Koch et al., 2007), which consists of cobalt/nickel-responsive transcriptional repressor RcnR (Iwig et al., 2008), cobalt/nickel transporter RcnA (Rodrigue et al., 2005), and RcnA-function-regulating protein RcnB (Bleriot et al., 2011). RcnA is in charge of transportation of cobalt/nickel ions and keeps their appropriate intracellular concentrations. In the absence of Co(II) or Ni(II), RcnR assembles into a homotetramer to prevent transcription of RcnA and RcnB by binding to the promoter region preceding *rcnA* (Iwig and Chivers, 2009). In the presence of Co(II) or Ni(II), RcnR dissociates from the promoter region so that the downstream genes can be expressed. The RcnR operator consists of two RcnR recognition motifs (TACT-G6-N-AGTA) (Iwig and Chivers, 2009). The concentration of Co(II)/Ni(II) is very low in municipal water; therefore, the components of the *rcn* operon have the potential to construct strict Co(II)/Ni(II)-induced gene expression systems.

In this study, we developed a tailor-made cobalt-induced expression system (CIES) in *E. coli* to overproduce highly active Co-NHase by coupling the Co-dependent induction and enzyme maturation through reprogramming the native *rcn* operon. It consisted of a strong promoter P_veg_ from *Bacillus subtilis* (Fu et al., 2006), Co- or Ni-responsive repressor RcnR, and the corresponding operator from *E. coli*. These biological parts from diverse bacteria were elaborately reprogrammed to design a sophisticated gene circuit that regulated the expression of a gene. By programming a cobalt homeostasis system, including cobalt efflux protein RcnA from *E. coli* and cobalt influx protein NiCoT from *Novosphingobium aromaticivorans*, the performance of CIES could be adjusted to desired levels. Cobalt ion in the CIES applied to Co-NHase production synchronically serves as both the inducer and the ligand for NHase. The scale-up in a 5-L fermenter verified that the synthetic CIES is a superior system for the production of Co-NHase compared to other commonly used systems, like the IPTG-induced expression system. More importantly, the design of CIES in this study addressed the feasibility and advantages of metal-induced expression systems for the production of distinct metalloenzymes.

## Materials and Methods

### Bacterial Strains, Plasmids, and Growth Conditions

All strains and plasmids used in this study were listed in Supplementary Table S1. Plasmid pET28a-GFP was manufactured by inserting the green fluorescent protein (GFP) gene, cloned from pBSG03, into pET28a. Plasmid pEV-GFP was constructed by substituting P_T__7__–lac_ with P_veg_. P_veg_ is a constitutive promoter from *B. subtilis* and possesses high transcriptional activity in both *B. subtilis* and *E. coli*. Plasmid pEVO-GFP was constructed by inserting the *rcnR* binding site (*rcnO*) between P_veg_ and a ribosome binding site (RBS). Another copy of the *rcnR* gene was inserted into pEVO-GFP, yielding pEVO-GFP-*rcnR*, to express sufficient repressors. For the construction of NHase production plasmid, GFP of appropriate plasmid was substituted with NHase coding sequences. Luria-Bertani (LB) medium (10 g/L tryptone, 5 g/L yeast extract, and 10 g/L NaCl, pH 7.0) was used for cell culture and expression of proteins. When solid media were prepared, 1.5% agar was used. The concentration of kanamycin for selection and growth is 50 μg/ml.

### Genetic Manipulation

In this study, all plasmid construction made use of the Gibson Assembly technique as described previously (Han et al., 2019). For short sequences’ insertion and substitutes, such as P_veg_ and *rcnO*, the target sequences were synthesized into corresponding primers. For clones of multiple DNA fragments, we amplified insert genes from templates and linearized vector backbones using appropriate primers and then ligated the amplified fragments *in vitro* using the Gibson Assembly depending on the homologous arms designed in primers formerly. The brief protocol to construct plasmid was as follows: (i) DNA fragments were amplified by polymerase chain reaction (PCR) with PrimeSTAR^®^ Max DNA Polymerase (Takara, Japan). (ii) The PCR product was treated by *Dpn*I to eliminate the template and then purified with the GeneJET Gel Extraction Kit (Thermo Fisher Scientific, United States). (iii) Purified DNA fragments (2.5 μl in total) were added into the 1.33 × Master Mix (7.5 μl). Then the mixture was incubated at 50°C for 30 min. (iv) The reaction liquid was transformed into component cells of *E. coli* JM109, and the cells were plated onto an LB agar plate with an appropriate antibiotic. All primers used for PCR were listed in Supplementary Table S2.

### GFP Expression and Fluorescence Assay

GFP served as the reporter to determine the biological function of the expression system. A single clone of *E. coli* strains harboring recombinant GFP expression plasmids was inoculated into test tubes containing 5 ml LB and cultured overnight at 37°C with shaking at 200 rpm. Then the seed liquid was transferred into a test tube or 250-ml shake flasks containing LB at the ratio of 1:50, after which the cultures were incubated at 37°C with shaking at 200 rpm. Cobalt chloride was added at 2 h after inoculation to induce the GFP gene expression. The cultures were periodically sampled over the culturing to monitor the growth and GFP expression. Cells were harvested by centrifugation at 8,000 rpm for 5 min, and the pellet was washed three times with phosphate-buffered saline (PBS, 8 g/L NaCl, 0.2 g/L KCl, 1.44 g/L Na_2_HPO_4_, and 0.24 g/L KH_2_PO_4_, pH 7.4) and resuspended in PBS with appropriate dilution. Each sample (200 μl) was transferred into 96-well black-walled plates and analyzed by PerkinElmer EnSpire^®^ 2300 Multimode Plate Reader (excitation 495 nm, emission 525 nm). For determination of specificity of metal and inductivity at various concentrations of metal, the GFP expression was carried out in test tubes.

### Expression of NHase and Enzymatic Activity Assay

In this study, a subunit-fused NHase with higher activity and thermostability was used (Xia et al., 2016). A single colony harboring NHase expression plasmid was placed into a test tube containing 5 ml of LB with kanamycin and then cultured overnight at 37°C with 200 rpm shaking. Then, 1 ml of cultured cells was transferred into a 250-ml shaking flask containing 50 ml of LB and grew at 37°C with 200 rpm shaking. After an appropriate concentration of cobalt chloride was added, the growth temperature dropped to 25°C to express NHase avoiding inclusion body formation.

For enzymatic activity determination, 10 μl of cultured cells was mixed with 490 μl of 200 mM 3-cyanopyridine solution (dissolved in 10 mM phosphate buffer, pH 7.4). The reaction was carried out at 26°C for 10 min and then stopped with the addition of 500 μl of acetonitrile. After filtration by a 0.22-μm filter and appropriate dilution with acetonitrile, the concentration of product nicotinamide was determined by high-performance liquid chromatography (HPLC). The mobile phase was a mixture solution of water and acetonitrile (2:1), and the flow rate was 0.6 ml/min. The amount of nicotinamide formed was determined by measuring the absorbance at 215 nm and extrapolation from a standard curve. One unit (U) of NHase activity is defined as the amount of enzyme needed for the production of 1 μmol of nicotinamide per minute under these assay conditions.

### Overproduction of NHase in a 5-L Fermenter

The initial medium for fermentation was 2 L of 2 × YT medium (16 g/L tryptone, 10 g/L yeast extract, and 5 g/L NaCl) containing 20 ml of trace elements [10 g/L FeSO_4_⋅7H_2_O, 3 g/L CuSO_4_⋅5H_2_O, 0.5 g/L MnSO_4_⋅4H_2_O, 5.25 g/L ZnSO_4_⋅7H_2_O, 0.1 g/L (NH_4_)Mo_7_O_24_, 0.23 g/L Na_2_B_4_O_7_⋅10H_2_O, and 2 g/L CaCl_2_]. The fed-batch medium consists of 500 g/L anhydrous glucose, 7.33 g/L MgSO_4_⋅7H_2_O, 4 g/L tryptone, and 4 g/L yeast extract. The inducer was 100 ml of a solution containing 10 g/L CoCl_2_6H_2_O. Seed culture was initiated by inoculating 40 ml of 2 × YT medium in a 250-ml shake flask with a single colony and then incubated for 12 h at 37°C with shaking at 200 rpm. The 5-L fermenter containing 2 L of the initial medium was inoculated with 40 ml of seed culture. The DO was kept at 30% by automatically adjusting agitation speed (200–1,000 rpm) and the air flow rate (4–7 L/min). The pH was kept at 7.0 automatically by adding NH_4_OH. When OD_600_ reached 7.0, fed-batch cultivation started with a speed of 9.6 ml/h to maintain a glucose concentration of 0.2–1.0 g/L. When OD_600_ reached 10.0, 100 ml of CoCl_2_ solution was fed with a speed of 10 ml/h, and the fermentation temperature was decreased to 24°C. For NHase production by pET system in a 5-L fermenter, the inducer IPTG with 0.4 mM of final concentration was fed along with the CoCl_2_ solution. Other fermentation conditions using the pET system were kept consistent with those using CIES.

## Results and Discussion

### Design, Construction, and Verification of CIES in *E. coli*

**FIGURE 1:**
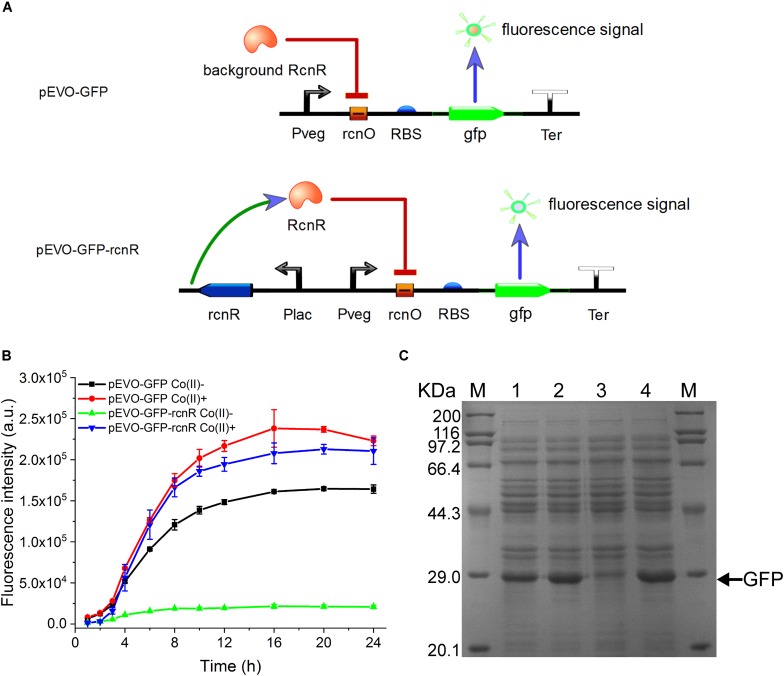
Construction and verification of CIES. **(A)** Schematic diagram of CIES. **(B)** GFP expression profile by cells harboring pEVO-GFP and pEVO-GFP-*rcnR*. Cobalt chloride was added at 2 h after inoculation with 100 μM of the final concentration. **(C)** SDS-PAGE analysis of GFP expression by cells harboring pEVO-GFP and pEVO-GFP-*rcnR*. M: marker. 1–4: pEVO-GFP Co(II)-, pEVO-GFP Co(II)+, pEVO-GFP-*rcnR* Co(II)-, and pEVO-GFP-*rcnR* Co(II)+. The band of GFP was indicated by an arrow. All error bars in figures presented root-mean-square deviation of three separate experiments.

It is very important to maintain homeostasis of heavy metal for bacteria adapting a diverse milieu. In *E. coli*, the *rcn* operon is employed to adjust the intracellular and extracellular concentrations of cobalt and nickel. This natural regulation pattern provides an ideal model to establish an artificially cobalt-induced system by employing the key components of the cobalt/nickel homeostasis system in *E. coli*. To construct a CIES, we initially used the native promoter P_rcnA_. However, the activity of P_rcnA_ was too low to efficiently express target protein (data not shown). Therefore, constructing CIES using combined elements was developed. At first, we constructed a hybrid promoter P_vco_ by fusing P_veg_, a strong constitutive promoter from *B. subtilis*, to the RcnR operator *rcnO* (Figure 1A and Supplementary Table S4). Theoretically, in the absence of cobalt, the activity of P_vco_ is able to be repressed by RcnR, while in the presence of cobalt, the hybrid promoter could be activated upon the dissociation of the repressor RcnR. To authenticate the designed function mediated by the hybrid promoter, we constructed plasmid pEVO-GFP harboring P_vco_ equipped with a strong RBS and GFP downstream. The fluorescent intensity of GFP was measured in the absence and presence of Co(II). Unexpectedly, the data exhibited that the fluorescent intensity against culture time was increased steadily either in the absence or in the presence of Co(II), although the overall fluorescent level in the presence of Co(II) was higher than that in its absence, indicating that the intrinsic expression of RcnR from chromosomes is insufficient to repress the activity of P_vco_ (Figures 1B,C).

To resolve this problem, we inserted an expression cassette harboring *rcnR* under the control of promoter P_lacI_ into pEVO-GFP, producing plasmid pEVO-GFP-*rcnR* (Figure 1A, lower panel). In this modified system, the fluorescent intensity of GFP in the absence of Co(II) was kept at a relatively low level over the entire culture process while the GFP intensity kept increasing to a high level during the culture in the presence of Co(II), suggesting that the synthetic CIES has gained a Co(II)-inducible function (Figure 1B). Moreover, SDS-PAGE analysis confirmed the highest expression level of GFP between the two synthetic CIES platforms in Figure 1C. In addition, we also evaluate the specificity of CIES to six other metal ions: Ca(II), Mg(II), Zn(II), Fe(II), Cu(II), and Mn(II). The result showed that the CIES can respond more specifically and efficiently to Co(II) than to Ni(II) (Supplementary Figure S1).

### Evaluation and Tuning of the Performance of CIES

**FIGURE 2:**
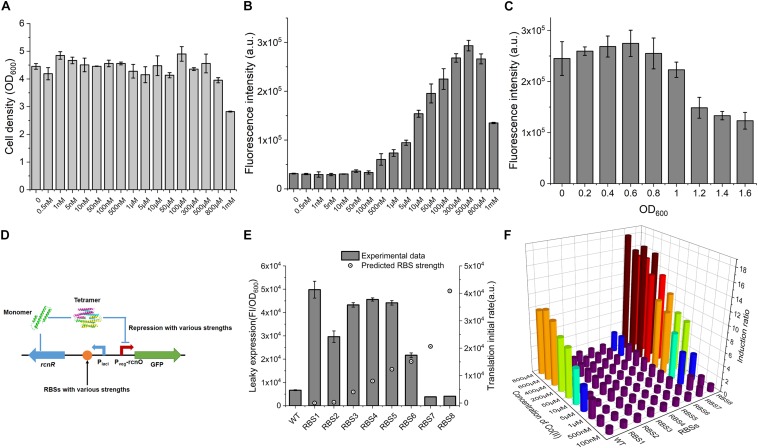
Characterization of CIES. **(A)** The influence of concentration of Co(II) on cell growth. **(B)** The influence of concentration of Co(II) on GFP expression. **(C)** The influence of induced occasion on GFP expression. **(D)** Schematic diagram of various repression strengths by various RcnR expression strengths. **(E)** The negative correlation between RcnR expression strength and leaky expression. **(F)** Determination of induction ratio under combinations of different RcnR expression strengths and different concentrations of Co(II). All error bars in figures presented root-mean-square deviation of three separate experiments. FI: Fluorescence intensity.

Excessive concentration of heavy metal was toxic to cells because it destroys the metal homeostasis (Chandrangsu et al., 2017). To ascertain the effect of Co(II) concentration on the cell growth so that we can determine a workable Co(II) concentration for CIES, here, the performance of CIES at various concentrations of Co(II) was tested. We firstly measured OD_600_ after treatment with a series of concentrations of Co(II). The results displayed that the concentrations of Co(II) from 0.5 nM to 500 μM were atoxic to the cell growth, except for that of 800 μM and 1 mM (Figure 2A), indicating that a broad range of inducer concentrations can be applied to this system. Accordingly, we determined the responsive profile to the series of concentrations set above. We found that the Fluorescence intensity (FI) of GFP increased steadily with Co(II) concentrations between 500 nM and 500 μM (Figure 2B). The range of valid Co(II) concentrations (<500 μM) for CIES covered the concentration we used in the IPTG-induced pET system (≈420 μM) for Co-NHase production (Xia et al., 2016). In addition, according to the results of OD_600_, we concluded that the toxicity of high concentrations of Co(II) (more than 500 μM) suppressed GFP expression. Meanwhile, we determined the most appropriate induction timing to maximize working efficiency. The data showed that when Co(II) induction began at the cell density (OD_600_) of 0.6, the expression of GFP reached the highest level compared to the other cell density points (Figure 2C).

The repressor RcnR functions as a homotetramer, and the multimerization rate is remarkably influenced by the concentration of monomers (Iwig et al., 2008) (Figure 2D and Supplementary Figure S2). We have revealed in this study that the level of repressor RcnR controls the responsive efficiency of the CIES. Therefore, we tune the expression level of RcnR by designing a series of synthetic RBSs with a continuum of predicted strength. To do this, eight synthetic RBSs (Supplementary Table S3) were designed using the bioinformatics tool RBS Calculator (Ahmad et al., 2014). Then they were inserted separately preceding the *rcnR* in the expression cassette (Figure 2D and Supplementary Table S3). We evaluated the tunable performance of RcnR by RBS through the leaky expression of GFP in the absence of Co(II). We observed that the leaky expression decreased along with the increasing predicted strength of RBS (Figure 2E). Then we combined the RBS-dependent tuning system with different concentrations of Co(II) to appraise the profile of the induction ratio (Supplementary Figure S3). We found from the plot that a high level of repression mediated by the high level of RcnR was prone to outputting a high induction ratio in the presence of Co(II) at high concentrations. Besides, when the systems were inducted by different concentrations of Co(II), the systems with high levels of RcnR (tuned by RBS7 and RBS8) had a regular induction ratio compared with that of others (Figure 2F). In each RcnR-level group, the higher concentration of Co(II) induced a stronger expression of GFP, resulting in a higher induction ratio. These results suggest that the functionality of the synthetic CIES is precisely regulated and readily tuned by synthetic RBS.

### Evolving CIES to Version 2.0 *via* Programming a Synthetic Cobalt Homeostatic Circuit

**FIGURE 3:**
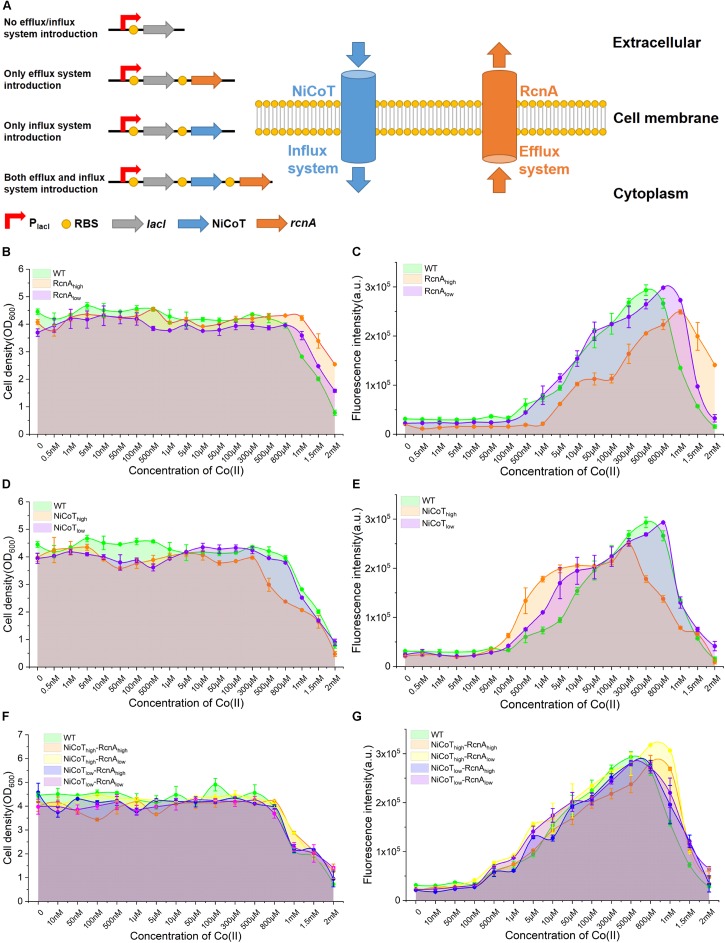
Characterization of the performance of CIES under synthetic cobalt homeostasis system. **(A)** Schematic diagram of synthetic cobalt homeostasis system by introducing the cobalt influx system (NiCoT) and/or efflux system (RcnA). **(B,C)** The influence of efflux system introduction on the cell growth and GFP expression of CIES at different concentrations of Co(II). **(D,E)** The influence of influx system introduction on the cell growth and GFP expression of CIES at different concentrations of Co(II). **(F,G)** The influence of both influx system and efflux system introduction on the cell growth and GFP expression of CIES at different concentrations of Co(II). All error bars in figures presented root-mean-square deviation of three separate experiments.

As a heavy metal ion, a high level of Co(II) in fermentation not only is toxic to *E. coli* cells but also is a kind of potential contamination to the environment and human beings. According to the results in this study, CIES can be effectively induced neither by less than 1 μM nor by more than 500 μM of Co(II) due to destructive cobalt homeostasis. We sought to broaden the available range of Co(II) for induction, through rebalancing the import and export of Co(II) by reconstitution of metal homeostasis. Metal homeostasis in bacteria comprises an efflux system for expelling metals and an influx system for uptake of metals. In this study, the cobalt transporters RcnA from *E. coli* and NiCoT from *N. aromaticivorans* (Duprey et al., 2014) were employed to design synthetic efflux and influx modules, respectively. The NiCoT from *N. aromaticivorans* has been reported to be better in helping *E. coli* take in Co(II) compared with NiCoT from other organisms (Duprey et al., 2014). In this system, *E. coli* cells were prone to uptake of Co(II) by NiCoT at low concentrations, while being prone to expelling Co(II) by RcnA to prevent toxicity at high concentrations (Figure 3A, the right panel).

We firstly dissected the performance of *E. coli* cell growth and induction only in the presence of RcnA. We constructed two plasmids derived from pEVO-GFP-*rcnR*, pEVO-GFP-*rcnA*(H), and pEVO-GFP-*rcnA*(L), harboring *rcnA* cloned downstream of *rcnR* combined with strong and weak synthetic RBSs, respectively, to regulate RcnA expression at a high level (RcnA_high_) and a low level (RcnA_low_) (Figure 3A, the left panel). We found that expression of the high level of RcnA (RcnA_high_) resulted in a higher density of host cells than the wild-type (WT) strain and low-RcnA-expressing cells (Figure 3B). However, although the induced expression of GFP of RcnA_high_ was higher than that of WT and RcnA_low_ at high levels of Co(II) (1.5 and 2 mM), the overall expression levels of GFP were lower than those in the other two systems (Figure 3C). These results manifest that overexpression of RcnA in CIES enables protection of the cells by adaptation to the high Co(II), as well as retention of higher induced expression levels at high levels of Co(II), but decreases the overall induced levels over a board range of concentrations.

Upon these results, we next explored the influence of enhancement of Co(II) import on cell growth and induction efficiency. We constructed two plasmids, pEVO-GFP-*NiCoT*(H) and pEVO-GFP-*NiCoT*(L), derived from pEVO-GFP-*rcnR* by insertion of *NiCoT* downstream of *rcnR* separately combined with strong and weak synthetic RBSs preceding the *NiCoT*, respectively, allowing high expression levels of NiCoT in the NiCoT_high_ system and low expression levels of NiCoT in the NiCoT_low_ system. Although cell growth of the NiCoT_high_ system was significantly suppressed in the presence of Co(II) of more than 300 μM, indicating that cells are more sensitive to Co(II) in NiCoT-introduced system, the GFP expression was able to be significantly induced to a higher level from 100 nM to 10 μM compared with that of WT and the NiCoT_low_ system (Figures 3D,E). Overall, these results confirmed that the cobalt efflux protein RcnA could alleviate toxicity by the high concentration of Co(II), while the cobalt influx protein NiCoT could enhance Co(II) uptake, resulting in the induction of GFP at low concentrations of Co(II).

The effectiveness of RcnA and NiCoT to tune the cell growth and induction level provided a prerequisite for design-programmable homeostasis of Co(II) in *E. coli* cells, which can combine the advantageous effect of the two proteins together in the CIES. On the basis of the concept, we introduced both RcnA and NiCoT together into *E. coli* cells and configured four combinatorial high and low expression levels between NiCoT and RcnA by synthetic RBSs, generating different expression combinations of NiCoT_high_–RcnA_high_, NiCoT_high_–RcnA_low_, NiCoT_low_–RcnA_high_, and NiCoT_low_–RcnA_low_ (Figure 3A, the left panel). Measurement of cell growth and induction profiles at different Co(II) concentrations displayed that the cells harboring NiCoT_high_–RcnA_high_ and NiCoT_high_–RcnA_low_ were well protected at the high levels of Co(II). Meanwhile, the induced levels of GFP at those concentrations were significantly higher than those of other combinations, as well as the WT (Figures 3F,G). These results suggest that the function of CIES evolves by reprogramming the homeostasis of Co(II) in *E. coli*, which enables the cells to be more adaptive under the milieu of high metal concentrations. A highly efficient induced system is achieved by the evolved CIES 2.0.

There are some reports focused on altering the resistance to or other properties of heavy metals in plants and bacteria through engineering metal homeostasis (Ovecka and Takac, 2014; Yoon et al., 2018). For example, interfering with the *znt* operon responsible for zinc homeostasis in *E. coli* is capable of modulating the properties of metal-sensing whole-cell bioreporters (Yoon et al., 2018). The introduction of NiCoT enhances the accumulation of Co(II) and Ni(II) in *E. coli* cells (Duprey et al., 2014). In this study, we addressed the feasibility of using a synthetic metal homeostatic circuit to augment the performance of a metal-induced expression system.

### Application of the CIES for Overproduction of Co-NHase

**FIGURE 4:**
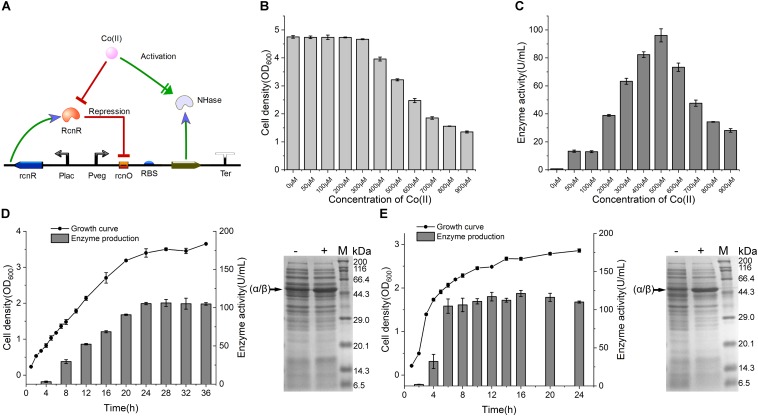
NHase production by CIES. **(A)** Schematic diagram of NHase production by CIES, in which Co(II) functioned as both inducer of expression and metal ligand of activated NHase. **(B)** The influence of concentration of Co(II) for NHase production on cell growth. **(C)** Determination of optimized concentration of Co(II) for NHase production. **(D)** Determination of growth curve and enzyme expression profile of NHase production by CIES over the fermentation process. **(E)** Determination of growth curve and enzyme expression profile of NHase production by IPTG-induced pET expression system over the fermentation process. -: no Co(II) added; +: added Co(II) with 500 μM of final concentration. The band of fused α/β subunit was indicated by an arrow. All error bars in figures presented root-mean-square deviation of three separate experiments.

To validate the applicability of CIES in production of Co-type metalloenzymes, we firstly employed CIES to express an engineered NHase (BA)P14K, which is a fusion mutant derived from the NHase of *P. putida* NRRL-18668 (Xia et al., 2016). Co(II) is a cofactor of (BA)P14K, which required formation of the active site, In CIES, the Co(II) diffused into the cells from the culture medium is simultaneously responsible for the induction as the inducer as well as for enzyme maturation as a cofactor (Figure 4A). We constructed a NHase-producing plasmid pEVO-(BA)P by substitution of the GFP sequence for (BA)P. Then we introduced the plasmid to *E. coli* JM109 cells, yielding the NHase-producing CIES. First of all, we determined the optimal Co(II) concentration used to produce (BA)P14K. The cell density and the enzyme activity of the NHase-producing cells after culturing with different concentrations of Co(II) were measured. The data showed that 500 μM Co(II) induced the highest enzymatic activity; however, inhibition of cell growth accordingly happened (Figures 4B,C). These results authenticated that Co(II) synchronically mediates induction and enzyme maturation of NHase.

To compare the productivity of NHase between the CIES and IPTG-induced system, we systematically monitored the cell growth and levels of produced activity profiles of NHase over culturing time. After induction by 500 μM Co(II) at 24°C for the CIES, the enzymatic activity reached 106.2 ± 4.6 U/ml (Figure 4D). Meanwhile, the highest production of the NHase after induction by 0.8 mM IPTG reached 121.4 ± 4.0 U/ml (Figure 4E). SDS-PAGE further confirmed the levels of NHase production between the CIES and IPTG-induced system (Figures 4D,E). The prominent advantage of the CIES platform, compared to the IPTG-induced system, is that it needs to introduce only one kind of molecule, Co(II), to accomplish induction and enzyme maturation, indicating that the production capability of CIES is a robust and highly efficient platform that is comparable to the generally regarded strong IPTG-induced system.

In *R. rhodochrous* J1, the high-molecular-mass NHase is induced by urea and Co(II) under the control of very complex regulatory systems. The mechanism of transcription repression of NHase by Co(II) in *R. rhodochrous* J1 was revealed recently (Lavrov et al., 2019). However, the mechanism of transcription activation by urea is still unclear (Komeda et al., 1996). Here, we heterologously expressed NHase in *E. coli* using CIES, which largely augments the production by simplifying the regulation.

### NHase Production by Co(II) Using CIES 2.0

**FIGURE 5:**
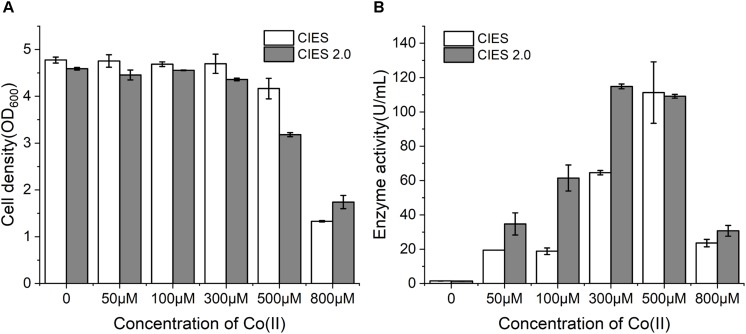
NHase expression by CIES and CIES 2.0. **(A)** Cell density of NHase expression strains at different concentrations of Co(II). **(B)** Enzyme activity of NHase expression strains at different concentrations of Co(II). All error bars in figures presented root-mean-square deviation of three separate experiments.

Cobalt is the essential ligand for active (BA)P14K. To further enhance the productivity and enzymatic activity, we employed CIES 2.0 to produce NHase. We constructed plasmid pEVO-(BA)P-NA(HL) derived from pEVO-GFP-NA by substitution of GFP for (BA)P14K, by which rebalancing of Co(II) was carried out by the high level of NiCoT and low level of RcnA. At working concentrations of Co(II), the cell density was similar between CIES and CIES 2.0. However, at an extraordinary concentration of Co(II) (800 μM), cells with the CIES 2.0 exhibited higher resistance to Co(II) (Figure 5A), confirming that the synthetic cobalt homeostatic circuit protects *E. coli* cells from Co(II) toxicity. Meanwhile, we found that the levels of enzymatic activity produced by the CIES 2.0 were higher than those produced by CIES at high levels of Co(II), except for the induction by 500 μM, which was equivalent between the two systems. The highest activity produced by the CIES 2.0 was 114.9 ± 1.4 U/ml, which was induced by 300 μM Co(II). This value was slightly higher than that in CIES induced by 500 μM Co(II) (111.2 ± 17.9 U/ml) (Figure 5B). These data elucidate that the CIES 2.0 is robust in the overproduction of Co-NHase by a broad range of Co(II) concentrations.

### Scale-Up Production of NHase by CIES 2.0

**FIGURE 6:**
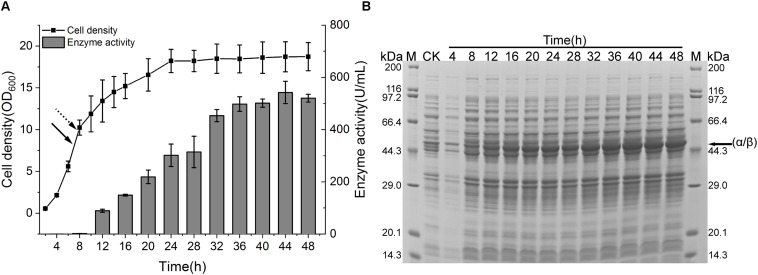
NHase production in a 5-L fermenter. **(A)** Cell growth and enzyme activity profiles of NHase-producing strain harboring pEVO-(BA)P-NA(HL) in a 5-L fermenter. The solid arrow indicated the start time of fed batch, and the dotted arrow indicated the time of adding Co(II). **(B)** SDS-PAGE assay of NHase production. CK means the same strain cultured in a test tube containing 2 × YT medium without addition of Co(II). The band of fused α/β subunit was indicated by the arrow.

To spur the application of CIES 2.0 to preparation of recombinant NHase at industrial scale, we performed a scale-up of the production of (BA)P14K by the CIES 2.0 in the 5-L fermenter. The strain harboring pEVO-(BA)P-NA(HL) was used in the scale-up system. When the OD_600_ reached 7 (after about 7 h), fed-batch cultivation started. We adopted exponential feeding to maintain the growth rate at a constant level. Induction by Co(II) began when the OD_600_ reached 10 (after 7.75 h) at the rate of 10 ml/h. The fermentation temperature was accordingly adjusted to 24°C (Figure 6A). After induction, the produced enzymatic activity gradually increased along with the induction progression until 36 h. The highest level of enzymatic activity was 542.2 ± 42.8 U/ml post 36 h of induction (Figure 6A). The SDS-PAGE analysis also verified the highly efficient production of NHase in the 5-L fermenter (Figure 6B). To appraise the superiority of CIES to the IPTG-inducible system, we scale up the NHase production by the pET system in the 5-L fermenter using the same fed-batch fermentation strategy as that of CIES 2.0. Compared with CIES 2.0, although the OD_600_ in the pET system reached over 30, the highest enzyme activity was only 446 ± 11.7 U/ml (Supplementary Figure S4). This indicates that the CIES 2.0 had advantages in the highly efficient production of active NHase.

In CIES 2.0, the expression and maturation of NHase were both controlled by Co(II), in which cells could automatically distribute the flow of Co(II). The scale-up of NHase production in the 5-L fermenter indicated that employing Co(II) as both an inducer and metal-ligand of NHase simplified fermentation conditions. In addition, high enzyme activity could be achieved using a relatively low concentration of Co(II) by the optimal cobalt homeostasis system. Therefore, the CIES 2.0 was low cost, environmentally friendly, and easy to scale up. More importantly, the development of a metal-induced gene expression system in this study provided a paradigm for developing a tailor-made expression system for metalloenzymes.

## Conclusion

In this study, a tailor-made CIES was developed for highly efficient production of Co-NHase. In CIES, Co(II) served as both an inducer of NHase expression and a cofactor of active NHase. A higher version of CIES 2.0 was also constructed from CIES through programming a cobalt efflux/influx system. As a benefit from efficient utilization of Co(II), high activity of NHase was achieved by adding less Co(II) using CIES 2.0. The scale-up production of NHase in a 5-L fermenter indicated that this system had potential in the industry. More importantly, we provided the feasibility and advantages of metal-induced expression systems for the production of distinct metalloenzymes.

Some types of Co-dependent enzymes have been overexpressed in *E. coli*, as well as the high-level synthesis of vitamin B12 in *E. coli* through metabolic engineering (Wu et al., 2017; Fang et al., 2018; Moon et al., 2018). In most cases of expressing and characterizing Co-dependent enzymes, the enzymes were first overexpressed and purified, then cobalt ions were added *in vitro*. According to the concentration of purified enzymes, 1 mM cobalt ions were usually needed. In this study, we provided a highly efficient expression system to directly express active Co-dependent enzymes *in vivo*. Through this way, we can easily evaluate the applied potential of enzymes, not just the properties of enzymes.

## Data Availability Statement

The authors declare that all the data supporting the findings of this study are available within the article and its Supplementary Information files or are available from the corresponding author on request.

## Author Contributions

LH and ZC conceived the project and designed the experiments. WC, QC, and KM performed the molecular cloning experiments. QL and FS measured the cell growth and GFP expression. ZC measured the NHase activity. WH and YW performed the scale-up experiments. LH, ZC, and ZZ analyzed the data and wrote the manuscript.

## Conflict of Interest

The authors declare that the research was conducted in the absence of any commercial or financial relationships that could be construed as a potential conflict of interest.
