# Supplementary material for: Efficient Overproduction of Active Nitrile Hydratase by Coupling Expression Induction and Enzyme Maturation via Programming a Controllable Cobalt-Responsive Gene Circuit
Source: Front Bioeng Biotechnol. 2020 Mar 24;8:193. doi: 10.3389/fbioe.2020.00193 (PMC7105576; doi:10.3389/fbioe.2020.00193)
Supplement: Supplementary file 1 [file Data_Sheet_1.docx]

Figure S1 Specificity of cobalt-induced system to divalent metal ions. Two level of concentrations (100 μM and 500 μM) of Co(II), Ni(II) and six divalent metal ions that often used in culture medium were employed to test the specificity of cobalt-induced system constructed in this study. To eliminate other interference, all metal ions were added as chlorine salt form. Error bars means the standard deviation of three three independent experiments.


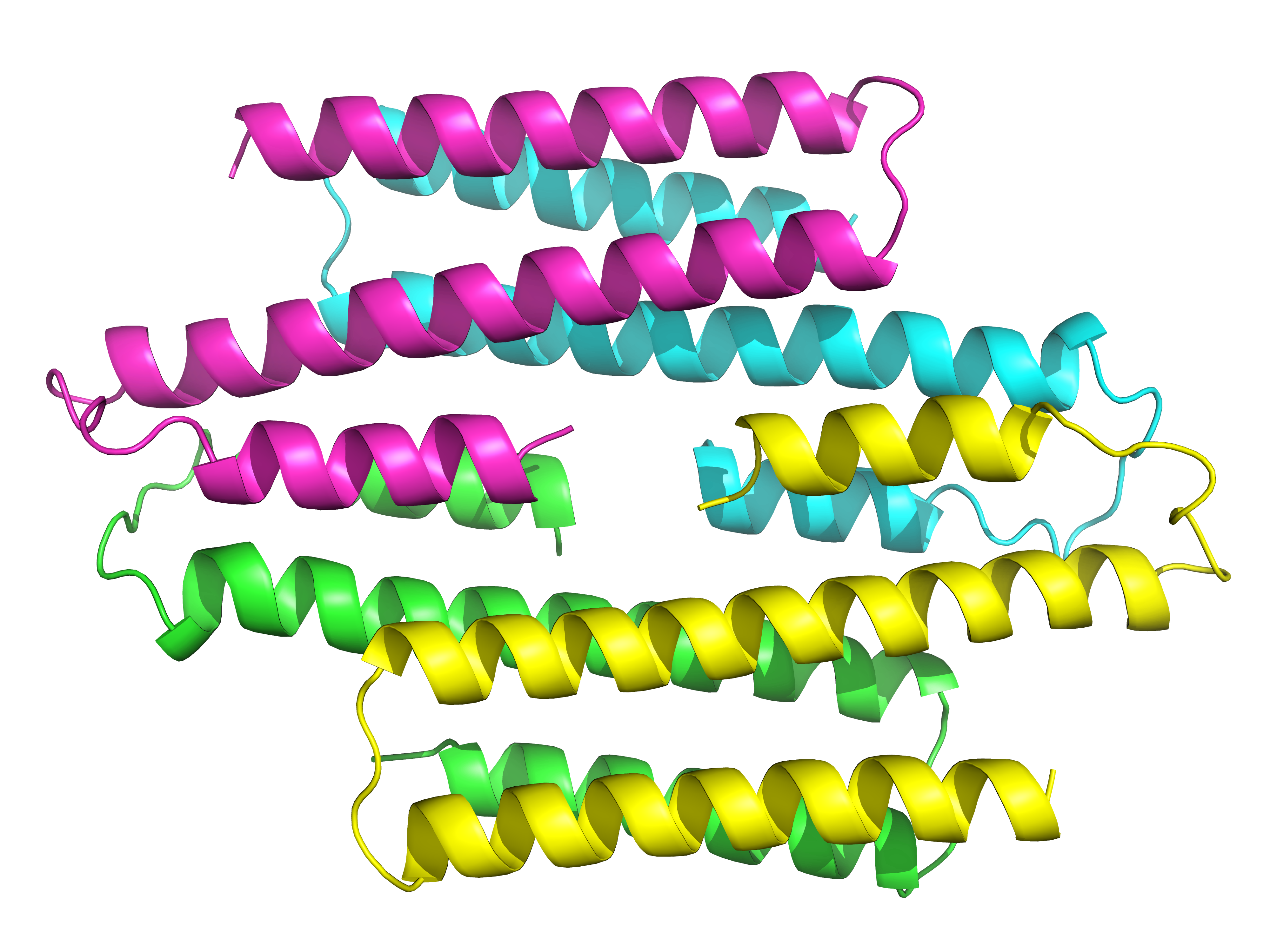


Figure S2 Homology modeling of RcnR. The 3D structure of RcnR was modelled by SWISS-MODEL using the FrmR (PDB code: 5LBM) from *E.coli* as template with identity of 41.57%. Four subunits were showed as green, yellow, cyan and magenta.


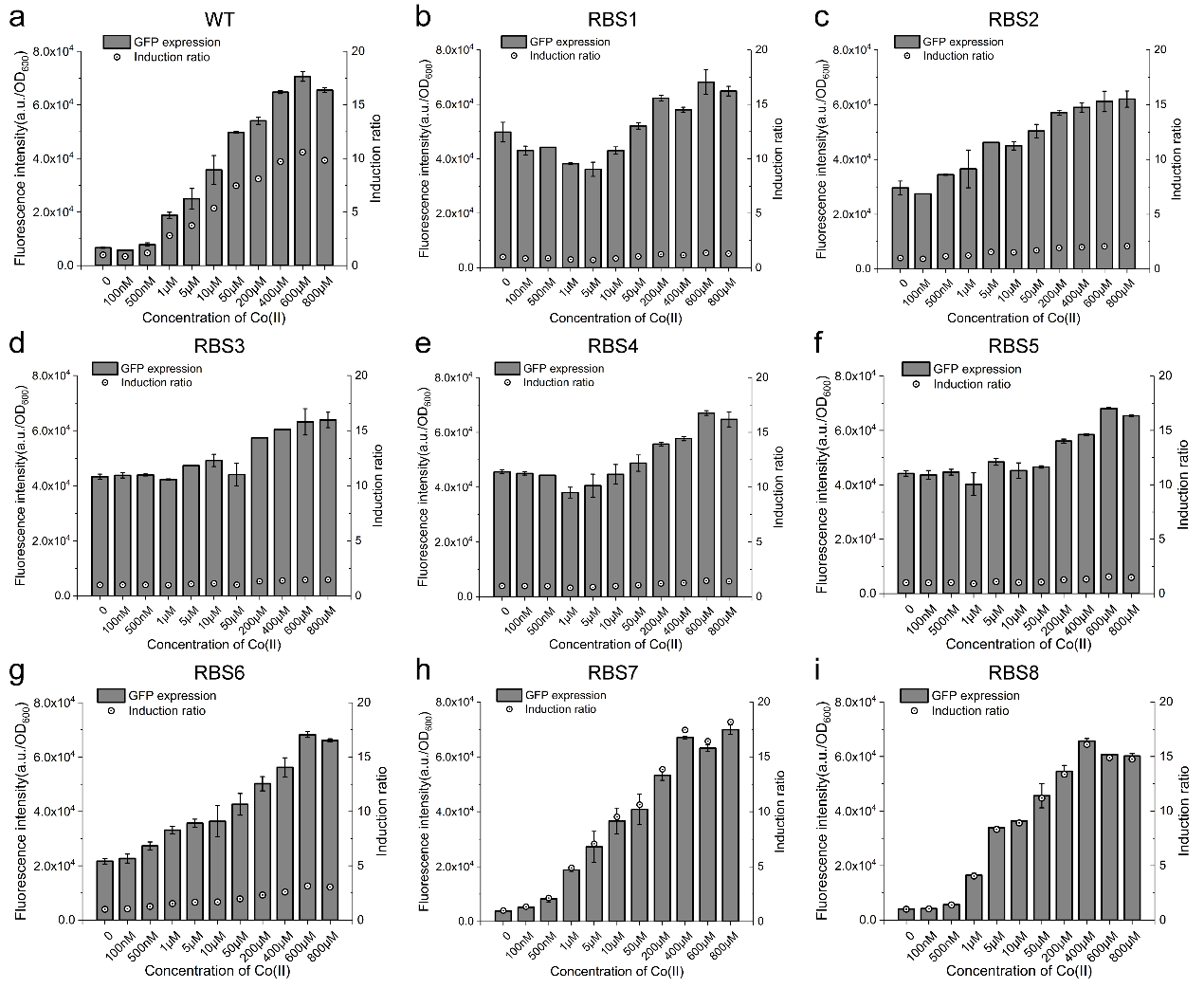


Figure S3 GFP expression under control of different RcnR expression level. The GFP expression and the corresponding induction ratio under different concentrations of Co(II) were represented as histogram and circle with a dot in the middle, respectively. Error bars means the standard deviation of three three independent experiments.


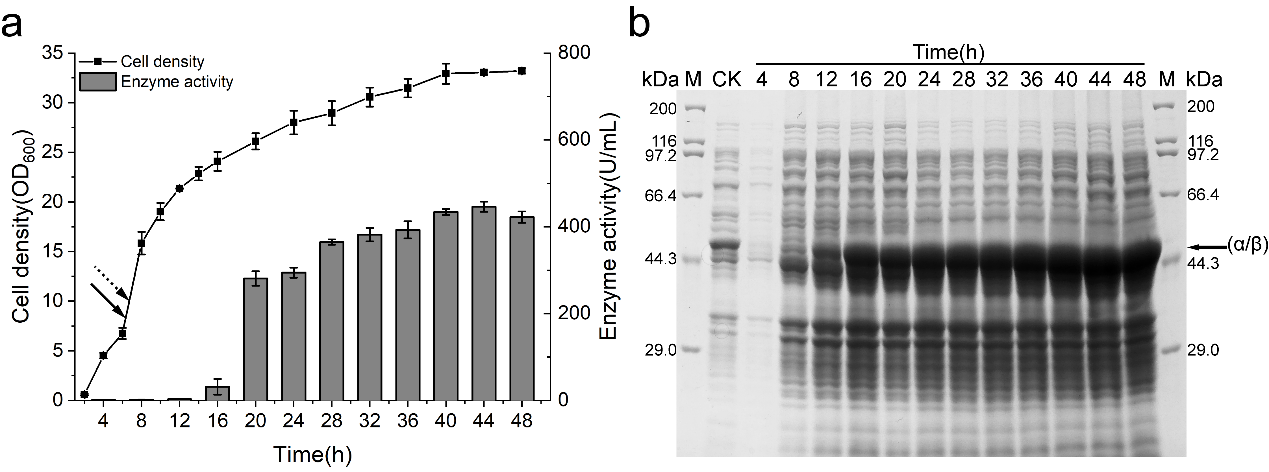


Figure S4 NHase production by pET expression system in 5-L fermentator. (a) Cell growth and enzyme activity profiles of NHase producing strain harboring pET28a-(BA)P14K in 5-L fermentator. The solid arrow indicated the start time of fed batch and the dotted arrow indicated the time of adding IPTG (0.4 mM of final concentration) and Co(II). (b) SDS-PAGE assay of NHase production. CK means the same strain cultured in test tube containing 2×YT medium without addition of Co(II). The band of fused α/β subunit was indicated by the arrow.

Table S1 Plasmids and strains

| Plasmids and strains | Relevant characteristics | References or source |
| --- | --- | --- |
| Plasmids |  |  |
| pET28a(+) | P_T7-lac_, KanR, ori | Lab stork |
| pBSG03 | *E.coli*-*B.subtilis* shuttle vector, P*_srfA_*, gfp, Amp^R^ in *Escherichia coli*, Kan^R^ in *Bacillus subtilis* | Lab stork^1^ |
| pET28a-(BA)P14K | Derived from pET28a(+), β/α subunit fused NHase was expressed by P_T7-lac_ | Lab stork^2^ |
| pET28a-GFP | Derived from pET28a(+), GFP was expressed by P_T7-lac_ | This study |
| pEV-GFP | Derived from pET28a-GFP, P_T7-lac_ was replaced with P_veg_ from *B.subtilis* | This study |
| pEVO-GFP | Derived from pEV-GFP, *rcnR* operator (rcnO) was inserted between P_veg_ and RBS | This study |
| pEVO-GFP-rcnR | Derived from pEVO-GFP, RBS-*rcnR* cassette was inserted into the downstream of lacI gene of plasmid | This study |
| pEVO-GFP-RBS_n_ | Derived from pEVO-GFP-rcnR, various synthetic RBSs were employed to tune *rcnR* expression | This study |
| pEVO-(BA)P | Derived from pEVO-GFP-rcnR, GFP was replaced with β/α subunit fused NHase | This study |
| pEVO-GFP-rcnA(H) | Derived from pEVO-GFP-rcnR, the gene cobalt efflux protein RcnA was expresse at high level with strong RBS | This study |
| pEVO-GFP-rcnA(L) | Derived from pEVO-GFP-rcnR, the gene cobalt efflux protein RcnA was expresse at low level with weak RBS | This study |
| pEVO-GFP-NiCoT(H) | Derived from pEVO-GFP-rcnR, the gene cobalt influx protein NiCoT from *Novosphingobium aromaticivorans* was expresse at high level with strong RBS | This study |
| pEVO-GFP -NiCoT (L) | Derived from pEVO-GFP-rcnR, the gene cobalt influx protein NiCoT from *Novosphingobium aromaticivorans* was expresse at low level with weak RBS | This study |
| pEVO-GFP-NA(HL) | Derived from pEVO-GFP-NiCoT(H), NiCoT and RcnA were expressed at high level and low level, respectively | This study |
| pEVO-GFP-NA(HH) | Derived from pEVO-GFP-NiCoT(H), both of NiCoT and RcnA were expressed at high level | This study |
| pEVO-GFP-NA(LH) | Derived from pEVO-GFP-NiCoT(H), NiCoT and RcnA were expressed at low level and high level, respectively | This study |
| pEVO-GFP-NA(LL) | Derived from pEVO-GFP-NiCoT(H), both of NiCoT and RcnA were expressed at low level | This study |
| pEVO-(BA)P-NA(HL) | Derived from pEVO-GFP-NA, GFP was replaced with β/α subunit fused NHase, NiCoT and RcnA were expressed at high level and low level, respectively | This study |
| Strains |  |  |
| *E.coli* JM109 | *recA*1, *supE*44 *endA*1 *hsdR*17 ( ^r-^k,m^+^ k) *gyrA*96 *relA*1 thi (*lac-proAB*) F’[*traD*36 *proAB*^+^ *lacI*^q^ *lacZ* ΔM15] | Lab stork |

Table S2 Primers

| Primers | Sequences (5’-3’) |
| --- | --- |
| Ppet-gfp-i1 | GTTTAACTTTAAGAAGGAGATATACCATGAGTAAAGGAGAAGAACTTTT |
| Ppet-gfp-i2 | GTTAGCAGCCGGATCTCATTTGTATAGTTCATCCATGCCATG |
| Ppet-gfp-v1 | CATGGCATGGATGAACTATACAAATGAGATCCGGCTGCTAAC |
| Ppet-gfp-v2 | AAAAGTTCTTCTCCTTTACTCATGGTATATCTCCTTCTTAAAGTTAAAC |
| Pev-1 | TGACAAAAATGGGCTCGTGTTGTACAATAAATGTAGGAATTGTGAGCGGATAAC |
| Pev-2 | GTACAACACGAGCCCATTTTTGTCAAATAAAATTTAAATTATATCAACGTTATTTCGCGGGATCGAG |
| PlacI-rcnR-i1 | TTAATTGCGTTGCGCTTATTTGATATATGAATCCAGCACC |
| PlacI-rcnR-i2 | TTCAGGGTGGTGAATATGTCTCATACAATCCGTG |
| PlacI-rcnR-v1 | GATTGTATGAGACATATTCACCACCCTGAATTG |
| PlacI-rcnR-v2 | CATATATCAAATAAGCGCAACGCAATTAATG |
| Pveg-rcnO-1 | GGGTAGTATCAGGTACTGGGGGGGAGTACCTCTAGAAATAATTTTGTTTAACTTTAAG |
| Pveg-rcnO-2 | TCCCCCCCAGTACCTGATACTACCCCCCAGTAGATACATTTATTGTACAACACGAGC |
| Plac-RBS1-1 | TCAATTAACTCTCTAAAAAATGTCTCATACAATCCGTG |
| Plac-RBS1-2 | AGACATTTTTTAGAGAGTTAATTGACTCTCTTCCGGG |
| Plac-RBS2-1 | TCAATTAATCACTCAAAAAATGTCTCATACAATCCGTG |
| Plac-RBS2-2 | AGACATTTTTTGAGTGATTAATTGACTCTCTTCCGGG |
| Plac-RBS3-1 | TCAATTAACCAGGTAAAAAATGTCTCATACAATCCGTG |
| Plac-RBS3-2 | AGACATTTTTTACCTGGTTAATTGACTCTCTTCCGGG |
| Plac-RBS4-1 | TCAATTAAGGCGGAAAAAAATGTCTCATACAATCCGTG |
| Plac-RBS4-2 | AGACATTTTTTTCCGCCTTAATTGACTCTCTTCCGGG |
| Plac-RBS5-1 | TCAATTAAGAGGTTAAAAAATGTCTCATACAATCCGTG |
| Plac-RBS5-2 | AGACATTTTTTAACCTCTTAATTGACTCTCTTCCGGG |
| Plac-RBS6-1 | TCAATTAATGGAGGAAAAAATGTCTCATACAATCCGTG |
| Plac-RBS6-2 | AGACATTTTTTCCTCCATTAATTGACTCTCTTCCGGG |
| Plac-RBS7-1 | TCAATTAAGGCGGTAAAAAATGTCTCATACAATCCGTG |
| Plac-RBS7-2 | AGACATTTTTTACCGCCTTAATTGACTCTCTTCCGGG |
| Plac-RBS8-1 | TCAATTAAGGGAGGAAAAAATGTCTCATACAATCCGTG |
| Plac-RBS8-2 | AGACATTTTTTCCTCCCTTAATTGACTCTCTTCCGGG |
| PNHase-i1 | GAAGGAGATATACCATGAATGGCATTCACGATACTG |
| PNHase-i2 | CAGCCGGATCTCAAGCCATTGCGGC |
| PNHase-v1 | GCCGCAATGGCTTGAGATCCGGCTGCTAAC |
| PNHase-v2 | GAATGCCATTCATGGTATATCTCCTTCTTAAAGTTAAAC |
| PRBS-high-2 | GGTCCTTATGTGGTTAATAACGTAGACTACGTTATTTGATATATGAATCCAGCACC |
| PRBS-low-2 | CGTCCTTATGTGGTTAATAACGTAGACTACGTTATTTGATATATGAATCCAGCACC |
| PRBS-high-rcnA-1 | ACGTTATTAACCACATAAGGACCGGTATTTTATGACCGAATTTACAACTCTTCTTC |
| PRBS-low-rcnA-1 | ACGTTATTAACCACATAAGGACGTGGATTTTATGACCGAATTTACAACTCTTC |
| PrcnR-rcnA-i2 | ATTGCGTTGCGCTTATCGCATTATGCCCATGAAGC |
| PrcnR-rcnA-v2 | ATAATGCGATAAGCGCAACGCAATTAATGTAAG |
| PRBS-high-NiCoT-1 | ACGTTATTAACCACATAAGGACCGGTATTTTATGCACCTGACCAAATCTCTG |
| PRBS-low- NiCoT-1 | ACGTTATTAACCACATAAGGACGTGGATTTTATGCACCTGACCAAATC TCTG |
| PrcnR-NiCot-i2 | AATTGCGTTGCGCTTAAGCACCAACAGAAACCTCG |
| PrcnR-NiCoT-v2 | GTTGGTGCTTAAGCGCAACGCAATTAATGTAAG |
| PNA-HH-2 | GGTCCTTATGTGGTTAATAACGTAGACTACGTTAAGCACCAACAGAAACCTCG |
| PNA-HL-2 | CGTCCTTATGTGGTTAATAACGTAGACTACGTTAAGCACCAACAGAAACCTCG |

Table S3 Sequences of synthetic RBSs for tuning RcnR expression

| RBS | Sequences (5’-3’) | Predicted initiation translation rate (au) |
| --- | --- | --- |
| RBS1 | TAACTCTCTAAAAA | 29.86 |
| RBS2 | TAATCACTCAAAAA | 299.07 |
| RBS3 | TAACCAGGTAAAAA | 4031.67 |
| RBS4 | TAAGGCGGAAAAAA | 8026.51 |
| RBS5 | TAAGAGGTTAAAAA | 12309.64 |
| RBS6 | TAATGGAGGAAAAA | 15136.7 |
| RBS7 | TAAGGCGGTAAAAA | 20652.67 |
| RBS8 | TAAGGGAGGAAAAA | 40876.33 |

Note: The variable sequences for RBS library construction were underlined.

Table S4 Nucleic acid sequences

| Name | Type | Sequences (5’-3’) |
| --- | --- | --- |
| GFP | Reporter | ATGAGTAAAGGAGAAGAACTTTTCACTGGAGTTGTCCCAATTCTTGTTGAATTAGATGGTGATGTTAATGGGCACAAATTTTCTGTCAGTGGAGAGGGTGAAGGTGATGCAACATACGGAAAACTTACCCTTAAATTTATTTGCACTACTGGAAAACTACCTGTTCCATGGCCAACACTTGTCACTACTTTCACTTATGGTGTTCAATGCTTTTCAAGATACCCAGATCATATGAAGCGGCACGACTTCTTCAAGAGCGCCATGCCTGAGGGATACGTGCAGGAGAGGACCATCTCTTTCAAGGACGACGGGAACTACAAGACACGTGCTGAAGTCAAGTTTGAGGGAGACACCCTCGTCAACAGGATCGAGCTTAAGGGAATCGATTTCAAGGAGGACGGAAACATCCTCGGCCACAAGTTGGAATACAACTACAACTCCCACAACGTATACATCACGGCAGACAAACAAAAGAATGGAATCAAAGCTAACTTCAAAATTAGACACAACATTGAAGATGGAAGCGTTCAACTAGCAGACCATTATCAACAAAATACTCCAATTGGCGATGGCCCTGTCCTTTTACCAGACAACCATTACCTGTCCACACAATCTGCCCTTTCGAAAGATCCCAACGAAAAGAGAGACCACATGGTCCTTCTTGAGTTTGTAACAGCTGCTGGGATTACACATGGCATGGATGAACTATACAAATGA |
| rcnR | Repressor | ATGTCTCATACAATCCGTGATAAACAGAAACTGAAAGCGCGTGCCAGTAAGATTCAGGGCCAGGTCGTGGCGCTCAAGAAAATGCTCGACGAGCCGCACGAATGCGCTGCAGTTTTACAACAGATTGCTGCTATCCGTGGCGCGGTAAACGGTCTGATGCGGGAAGTGATTAAAGGTCATCTGACGGAACACATCGTTCACCAGGGGGATGAGCTAAAACGTGAAGAAGATCTGGATGTCGTTCTGAAGGTGCTGGATTCATATATCAAATAA |
| (BA)P | Nitrile hydratase | Fusion subunit of α and β (BA)  ATGAATGGCATTCACGATACTGGCGGAGCACATGGTTATGGGCCGGTTTACAGAGAACCGAACGAACCCGTCTTTCGCTACGACTGGGAAAAAACGGTCATGTCCCTGCTCCCGGCGCTGCTCGCCAACGGCAACTTCAACCTCGATGAATTTCGGCATTCGATCGAGCGAATGGGCCCGGCCCACTATCTGGAGGGAACCTACTACGAACACTGGCTTCATGTCTTTGAGAACCTGCTGGTCGAGAAGGGTGTGCTCACGGCCACGGAAGTCGCGACCGGCAAGGCTGCGTCTGGCAAGACGGCGACGCCGGTGCTGACGCCGGCCATCGTGGACGGACTGCTCAGCACCGGGGCTTCTGCCGCCCGGGAGGAGGGTGCGCGGGCGCGGTTCGCTGTGGGGGACAAGGTTCGCGTCCTCAACAAGAACCCGGTGGGCCATACCCGCATGCCGCGCTACACGCGGGGCAAAGTGGGGACAGTGGTCATCGACCATGGTGTGTTCGTGACGCCGGACACCGCGGCACACGGAAAGGGCGAGCACCCCCAGCACGTTTACACCGTGAGTTTCACGTCGGTCGAACTGTGGGGGCAAGACGCTTCCTCGCCGAAGGACACGATTCGCGTCGACTTGTGGGATGACTACCTGGAGCCAGCGCCAGGTGGGCAATCACACACGCATGACCACCATCACGACGGGTACCAGGCACCGCCCGAAGACATCGCGCTGCGGGTCAAGGCCTTGGAGTCTCTGCTGATCGAGAAAGGTCTTGTCGACCCAGCGGCCATGGACTTGGTCGTCCAAACGTATGAACACAAGGTAGGCCCCCGAAACGGCGCCAAAGTCGTGGCCAAGGCCTGGGTGGACCCTGCCTACAAGGCCCGTCTGCTGGCAGACGGCACTGCCGGCATTGCCGAGCTGGGCTTCTCCGGGGTACAGGGCGAGGACATGGTCATTCTGGAAAACACCCCCGCCGTCCACAACGTCTTCGTTTGCACCTTGTGCTCTTGCTACCCATGGCCGACGCTGGGCTTGCCCCCTGCCTGGTACAAGGCCGCGCCCTACCGGTCCCGCATGGTGAGCGACCCGCGTGGGGTTCTCGCGGAGTTCGGCCTGGTGATCCCCGCCAACAAGGAAATCCGCGTCTGGGACACCACGGCCGAATTGCGCTACATGGTGCTGCCGGAACGGCCCGCGGGAACTGAAGCCTACAGCGAAGAACAACTGGCCGAACTCGTTACCCGCGATTCGATGATCGGCACCGGCCTGCCCACCCAACCCACCCCATCTCATTGA  Activator (P)  ATGAAAGACGAACGGTTTCCATTGCCAGAGGGTTCGCTGAAGGACCTCGATGGCCCTGTGTTTGACGAGCCTTGGCAGTCCCAGGCGTTTGCCTTGGTGGTCAGCATGCACAAGGCCGGTCTCTTTCAGTGGAAAGACTGGGCCGAGACCTTCACCGCCGAAATCGACGCTTCCCCGGCTCTGCCCGGCGAAAGCGTCAACGACACCTACTACCGGCAATGGGTGTCGGCGCTGGAAAAGTTGGTGGCGTCGCTGGGGCTTGTGACGGGTGGAGACGTCAACTCGCGCGCACAGGAGTGGAAACAGGCCCACCTCAACACCCCACATGGGCACCCGATCCTGCTGGCCCATGCGCTTTGCCCGCCAGCGATCGACCCCAAGCACAAGCACGAGCCACAACGCTCACCGATCAAGGTCGTTGCCGCAATGGCTTGA |
| rcnA | Efflux system for nickel and cobalt | ATGACCGAATTTACAACTCTTCTTCAGCAAGGAAACGCCTGGTTCTTCATCCCCAGCGCCATCTTACTTGGTGCGCTTCATGGCCTGGAACCAGGGCACTCAAAAACGATGATGGCGGCGTTTATCATCGCCATCAAAGGCACCATTAAACAAGCGGTGATGCTCGGACTGGCAGCAACTATTTCGCATACCGCAGTGGTCTGGTTAATTGCCTTTGGCGGGATGGTGATCAGCAAGCGCTTTACTGCTCAATCAGCAGAACCGTGGCTCCAGCTGATTTCCGCAGTGATCATTATTAGCACCGCGTTCTGGATGTTCTGGCGTACCTGGCGCGGCGAACGCAACTGGCTGGAGAATATGCACGGGCATGATTATGAGCATCATCATCACGATCACGAACATCACCACGACCATGGACATCATCACCATCACGAACATGGCGAGTATCAGGATGCCCATGCACGAGCCCATGCCAATGACATTAAACGACGCTTTGATGGTAGAGAGGTCACCAACTGGCAAATTTTGTTATTTGGCTTAACCGGTGGCCTTATCCCCTGCCCGGCAGCAATTACCGTGCTGTTGATATGCATTCAGTTGAAAGCCCTGACACTGGGCGCAACACTGGTCGTCAGTTTCAGCATTGGCCTGGCGTTAACGCTTGTCACCGTAGGCGTTGGCGCAGCAATCAGCGTTCAGCAGGTCGCAAAACGCTGGAGCGGATTTAACACTCTCGCTAAACGCGCCCCCTATTTTTCCAGTCTGTTGATTGGCTTAGTCGGTGTGTATATGGGCGTACATGGCTTCATGGGCATAATGCGATAA |
| NiCoT^3^ | Influx system for nickel and cobalt | ATGCACCTGACCAAATCTCTGCCGTCTCTGTCTCTGCGTCGTCGTATCGGTGCTCTGTTCGCTGGTCTGATCGCTGCTAATATAGGTGTCTGGGTCTGGGCTTTCTCTCTGTTCCACGCTCAGCCGCTGATGCTGGGTACCGCTGTTCTGGCTTGGGGTCTGGGTCTGCGTCACGCTGTTGACGCTGACCACATCGCTGCTATCGACAACGTTACCCGTAAACTGATGCAGGACGGTCAGAGGCCGGTGAGCGTTGGTTTCTGGTTCGCTATCGGTCACTCTGGTATCATCGCTATCGCTTCTATCATCATCGCTGTTACCGCTTCTGCTCTGTCTCAGTTCGGTGCTTTCAAAGAAATCGGTGGTGTTATCGCTACCGTTATCTCTGCTCTGTTCCTGTTCACCATCGCTGGTATGAACCTGGTTATCCTGCGTTCTGTTTGGCGTACCTTCGCTCACGTTCGTGCTGGTGGTTCTTACGCTGAAGACGACCTGGACCTGCTGCTGGGTGGTCGTGGTCTGCTGTCTCGTCTGTTCCGTCCGATGTTCCGTCTGGTTAACAAATCTTGGCACATGGCTCCGCTGGGTTTCCTGTTCGGTCTGGGTTTCGACACCGCTACCGAAGTTGCTATCCTGGGTATGTCTGCTTCTCAGGCTGCTGACGGTCTGTCTATCGGTACCATCCTGGTTCTGCCGGCTCTGTTCGCTGTTGGTATGGCTCTGATCGACACCGCTGACGGTGTTGTTATGCTGGGTGCTTACGAATGGGCTTTCGTTAAACCGATCCGTAAACTGTACTACAACATCACCATCACCCTGATCTCTGCTATCGTTGCTATCGTTATCGGTGGTATCGAAACCCTGGCTCTGATCGGTGACAAACTGGCTCTGACCGGTGCTCCGTGGCGTATCGCTATCGAACTGGGTGAAAACTTCAACGGTCTGGGTTTCGCTATCATCGGTCTGTTCGTTTTCTGCTGGCTGGCTTCTTACGCTATCTACCGTTGGAAGAGGTTCGACGAAATCGAGGTTTCTGTTGGTGCTTAA  The coding sequence of NiCoT was optimized for expression in *E. coli*. |
| P_veg_ | Promoter | AACGTTGATATAATTTAAATTTTATTTGACAAAAATGGGCTCGTGTTGTACAATAAATGTA |
| rcnO | rcnR binding site | TACTGGGGGGTAGTATCAGGTACTGGGGGGGAGTA |
| RBS_strong_ | Strong RBS for rcnA/NiCoT expression | CGTAGTCTACGTTATTAACCACATAAGGACCGGTATTTT |
| RBS_weak_ | Weak RBS for rcnA/NiCoT expression | CGTAGTCTACGTTATTAACCACATAAGGACGTGGATTTT |

**References**

1. Guan, C.; Cui, W.; Cheng, J.; Zhou, L.; Liu, Z.; Zhou, Z., Development of an efficient autoinducible expression system by promoter engineering in Bacillus subtilis. *Microbial cell factories* **2016,** *15*, 66.

2. Xia, Y.; Cui, W.; Liu, Z.; Zhou, L.; Cui, Y.; Kobayashi, M.; Zhou, Z., Construction of a subunit-fusion nitrile hydratase and discovery of an innovative metal ion transfer pattern. *Scientific reports* **2016,** *6*, 19183.

3. Duprey, A.; Chansavang, V.; Fremion, F.; Gonthier, C.; Louis, Y.; Lejeune, P.; Springer, F.; Desjardin, V.; Rodrigue, A.; Dorel, C., "NiCo Buster": engineering E. coli for fast and efficient capture of cobalt and nickel. *Journal of biological engineering* **2014,** *8*, 19.
